# Supplementary material for: Adult Neurogenesis Reconciles Flexibility and Stability of Olfactory Perceptual Memory
Source: bioRxiv. 2024 Nov 20:2024.03.03.583153. Preprint. [Version 3] doi: 10.1101/2024.03.03.583153 (PMC11087939; doi:10.1101/2024.03.03.583153)
Supplement: Supplementary file 1 [file NIHPP2024.03.03.583153v3-supplement-1.pdf]

## Supplementary information

## Discriminability

In addition to using a connectivity-based learning measure, we use an activity-based learning measure to characterize to what extent learning enhances the ability of downstream cortical neurons to discriminate between the odors based on their read-out of the MC activities. Because the MC rate model does not include any fluctuations in activity that would limit discriminability, we assume that the rates represent the mean values of independent Poisson spike trains for which the variance is given by their mean. We assume a linear read-out of the MC activities with the weights chosen optimally and characterize the discriminability of stimuli $A$ and $B$ in terms of the optimal Fisher discriminant $F_{opt}$ ***Adams et al.*** (***2019***),

$$F_{opt} = \sum_{i=1}^{N_{MC}} \frac{(M_i^{(A)} - M_i^{(B)})^2}{M_i^{(A)} + M_i^{(B)}}. \tag{1}$$

Thus, it can be seen that $F_{opt}$ will increase with the addition of MCs, reflecting the fact that even poorly discriminating MCs provide some additional information about the odors.

bioRxiv preprint doi: https://doi.org/10.1101/2024.03.03.583153; this version posted November 20, 2024. The copyright holder for this preprint (which was not certified by peer review) is the author/funder, who has granted bioRxiv a license to display the preprint in perpetuity. It is made available under aCC-BY 4.0 International license.

To verify that our connectivity-based measure of memory aligns with the function of the OB, we calculated the time course of the Fisher discriminant using the data that generated the results in Figure 2C (Figure S1B). Indeed, both measures yield qualitatively similar results, with the fast network learning and forgetting quickly, the slow network learning and forgetting slowly, and the age-dependent network learning quickly and forgetting slowly. Likewise, the neurogenic and non-neurogenic networks performed similarly.

In this study, we focused on the changes in the network connectivity rather than changes in MC activity. We therefore assessed the behavior of the system mostly in terms of the connectivity-based memory. This measure for the memory is agnostic with respect to the odor code, i.e. it does not depend on the type of read-out of the OB activity used by the animal (e.g. rate-based or timing-based ***Wilson et al.*** (***2017***); ***Bolding and Franks*** (***2017***)).

## Comparison with other methods resolving the flexibility-stability dilemma

Previous theoretical work has established a general framework in order to track the memory of an arbitrary stimulus in a stream of random uncorrelated stimuli based only on the properties of the network, without explicitly modeling neuronal activity. It has been used to evaluate models that confront the flexibility-stability dilemma ***Fusi et al.*** (***2005***); ***Roxin and Fusi*** (***2013***); ***Benna and Fusi*** (***2016***); ***Fusi*** (***2021***). In networks with $N$ simple synapses where plasticity occurs on a uniformly fast time scale, the initial memory grows as $\sqrt{N}$ while overall memory capacity grows only logarithmically with $N$ ***Amit and Fusi*** (***1994***); ***Fusi and Abbott*** (***2007***). Meanwhile, the complex synapses of the cascade model ***Fusi et al.*** (***2005***) and the bidirectional cascade model ***Benna and Fusi*** (***2016***) as well as the heterogeneity and structure of the partitioned memory system model ***Roxin and Fusi*** (***2013***) have been shown to allow the network to achieve far greater capacity. In the case of the cascade model and the partitioned memory system model, memory capacity on the order of $\sqrt{N}$ can be achieved, and in the case of the bidirectional cascade model memory capacity on the order of $N$ can be achieved, though the latter requires a great degree of complexity in the synapses.

To understand the scaling properties of our model and how they compare with other models, we situated it within this framework. More specifically, we consider a network initially with $N$ binary synapses. At each time step, each synapse is independently presented with a plasticity event, which attempts to flip the synapse depending on the presented stimulus and is accepted with probability $q_i$, the plasticity rate of synapse $i$ (Figure S6A). To quantify memory performance, we tracked the signal-to-noise ratio (SNR) of a single arbitrary stimulus previously encoded by the network (Figure S6B). We report the flexibility as the SNR immediately after stimulus presentation (the "initial memory" SNR(0)). The stability we characterize in terms of the time $T$ that it took for the SNR to decay to the value of 1 due to the storage of subsequent memories (the "memory lifetime"), which can be interpreted as the memory capacity. The presented stimuli are random and uncorrelated. Thus, on average the initial memory signal $\mu_i(t=0)$ of a stimulus associated with synapse $i$ is $q_i$ and the total initial memory signal $\mu(0)$ of the network is

$$\mu(0) = \sum_{i=1}^{N} \mu_i(0) = \sum_{i=1}^{N} q_i. \tag{2}$$

Meanwhile, as this is a system of binomially distributed variables, the variance of the signal can be roughly approximated as $\sqrt{N}$ ***Roxin and Fusi*** (***2013***), leading to a signal-to-noise ratio $SNR(0)$ of

$$SNR(0) = \frac{1}{\sqrt{N}} \sum_{i=1}^{N} q_i. \tag{3}$$

As ***Roxin and Fusi*** (***2013***) show, the dynamics of the signal to noise ratio of the memory can be described by

$$\frac{dSNR}{dt} = \frac{1}{\sqrt{N}} \sum_{i=1}^{N} \frac{d\mu_i}{dt} \tag{4}$$

bioRxiv preprint doi: https://doi.org/10.1101/2024.03.03.583153; this version posted November 20, 2024. The copyright holder for this preprint (which was not certified by peer review) is the author/funder, who has granted bioRxiv a license to display the preprint in perpetuity. It is made available under aCC-BY 4.0 International license.

where the $\mu_i(t)$ follow the equations

$$\frac{d\mu_i}{dt} = -q_i \mu_i. \tag{5}$$

To incorporate the key element of our plasticity model, we extend this model by making the plasticity rates $q_i$ depend on the ages of the cells such that

$$q_i = \begin{cases} q^{fast} & \text{if age of GC } i \text{ is} \leq T_c \text{ days} \\ q^{slow} & \text{if age of GC } i \text{ is} > T_c \text{ days,} \end{cases} \tag{6}$$

where $q^{fast} \gg q^{slow}$ and $T_c$ is the duration of the critical period during which the synapse is highly plastic. Following ***Fusi et al.*** (***2005***), we choose $q^{fast} \sim \mathcal{O}(1)$ and $q^{slow} \sim \mathcal{O}(N^{-\frac{1}{2}})$. If, at the time of the stimulus presentation, the fraction of synapses on young GCs is $k$, then according to Eq.3 the $SNR$ of that memory is given by

$$SNR(0) = \sqrt{N}(kq^{fast} + (1-k)q^{slow}). \tag{7}$$

If $kq^{fast} \gg q^{slow}$, the initial memory is controlled by the fast plasticity rate, $SNR(0) \sim \sqrt{N}kq^{fast}$. Indeed, in the rat brain the total number of GCs is a few million, while roughly 10,000 more are born on each day ***Kaplan et al.*** (***1985***). Since the critical period lasts about 14 days, about 140,000 GCs have enhanced plasticity, so $k$ is on the order of 0.1. Thus assuming $N \sim 10^8$, $kq^{fast} \approx 10^{-1} \gg q^{slow} \approx 10^{-4}$ is a valid assumption.

The memory duration is determined by the time $T$ at which $SNR(t)$ falls below some fixed threshold $\theta_{SNR}$. Solving Eqs.3-6, we have that for $t \geq T_c$, $SNR(t)$ is given by

$$SNR(t) = \left[ q^{slow}(1-k)\sqrt{N} + q^{fast}\frac{k\sqrt{N}}{T_c}\sum_{j=0}^{T_c} e^{(q^{slow}-q^{fast})j} \right] e^{-q^{slow}t}. \tag{8}$$

We use this to solve for the memory duration $T$ where $SNR(T) = \theta_{SNR}$. Again, using $q^{slow} \sim \mathcal{O}(\frac{1}{\sqrt{N}})$ and $q^{fast} \sim \mathcal{O}(1)$ we get

$$T = \sqrt{N}\log\left[\frac{1-k}{\theta_{SNR}} + \frac{k\sqrt{N}}{T_c\theta_{SNR}}\sum_{j=0}^{T_c} e^{(\frac{1}{\sqrt{N}}-1)j}\right]. \tag{9}$$

For large $N$, memory duration scales approximately as $T \sim \mathcal{O}(\sqrt{N}\log(\sqrt{N}))$, where the leading $\sqrt{N}$ arises from the inverse of $q^{slow}$. This shows that while initial memory is controlled by the fast plasticity of immature synapses, memory duration is controlled by the slow plasticity rate of mature synapses.

We verified these results computationally. First we compared the memory decay of our model to those of homogeneous models as well as the cascade model and the partitioned memory system model for a network of approximate size to the rat OB (Figure S6C). We show our model (red) has a similar initial memory and memory duration as the cascade model (blue) and the partitioned memory system model (green), all of which far outpace the initial memory of the slow-synapse model (grey) and the memory duration of the fast-synapse model (black). Notably, this reiterates that the increased memory capacity provided by neurogenesis is due to the age-dependent properties of adult-born neurons rather than the addition of neurons alone, and that this age-dependence can most efficient utilize the new synapses provided by adult neurogenesis.

Finally, we examine how the initial memory and the memory duration scale with $N$ (Figure S6D). We confirm that both the initial memory (Figure S6E) as well as the memory lifetime approximately follow $\sqrt{N}$ (Figure S6F). Thus, like the cascade model and the partitioned-memory model, our age-dependent model robustly resolves the plasticity-flexibility dilemma, simultaneously achieving the greatest initial memory and memory duration possibly afforded by the homogeneous network with constant plasticity.

bioRxiv preprint doi: https://doi.org/10.1101/2024.03.03.583153; this version posted November 20, 2024. The copyright holder for this preprint (which was not certified by peer review) is the author/funder, who has granted bioRxiv a license to display the preprint in perpetuity. It is made available under aCC-BY 4.0 International license.

998 ## Supplementary Figures

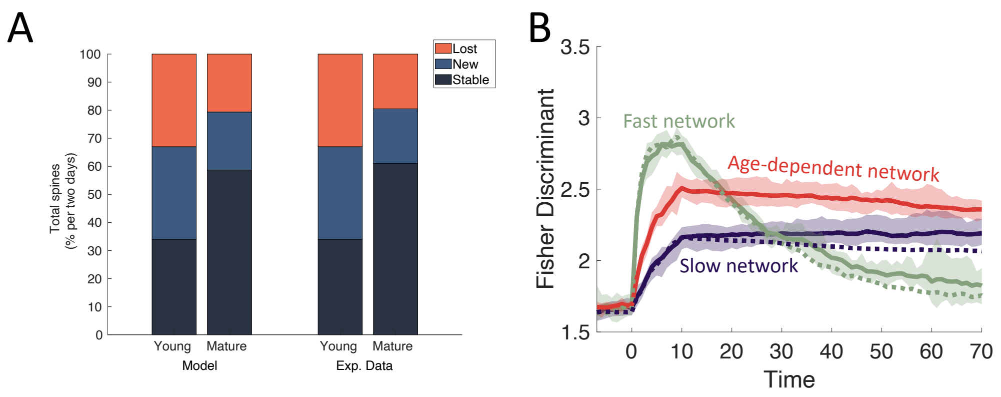


**Figure S1. (Related to Figure 2) Spine turnover and consistency of memory measure.** (A) Parameters governing spine turnover were fit so that the two day spine turnover rates in young and mature abGCs matched those previously reported in ***Sailor et al.*** (***2016***). (B) Odor-discriminability as characterized by the Fisher discriminant (Supplementary Information “Discriminability") exhibits the same behavior as the connectivity-based memory shown in Figure 2C.

bioRxiv preprint doi: https://doi.org/10.1101/2024.03.03.583153; this version posted November 20, 2024. The copyright holder for this preprint (which was not certified by peer review) is the author/funder, who has granted bioRxiv a license to display the preprint in perpetuity. It is made available under aCC-BY 4.0 International license.

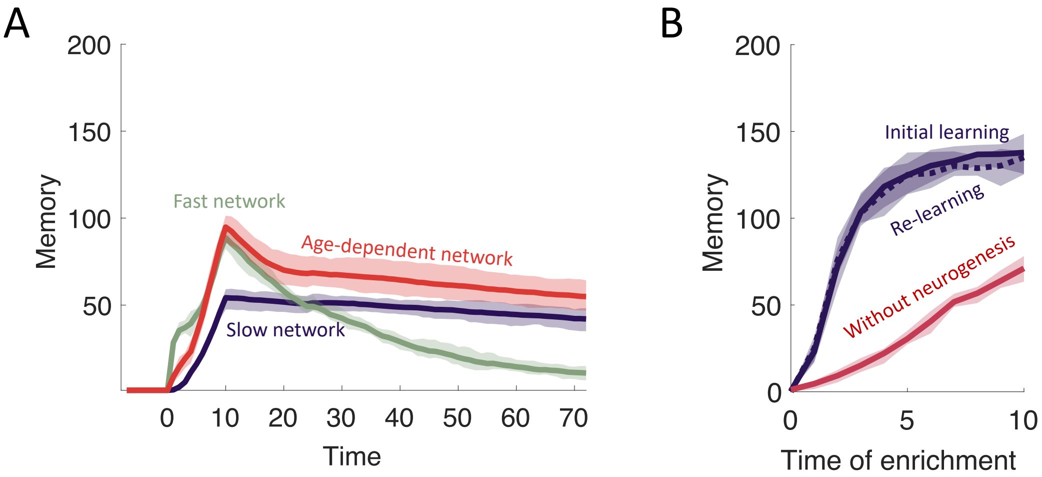


**Figure S2. (Related to Figure 3) Dependence of memory on dendritic development** (A) The same memory measurements were taken as in Fig.2C for the model with sensory-dependent dendritic development as well as increased excitability. The results are similar to those in Fig.2E, although the shifted birth-date dependence of abGC recruitment (Figure 3C) means that odor-encoding GCs are still in their critical period at the end of enrichment, leading to a short period of rapid memory decay. (B) Repeating the simulation in Figure 3D,F without sensory-dependent dendritic development. Relearning is no longer faster than the initial learning and is especially slow when neurogenesis is blocked. Here, $N_{conn} = 100$ to allow the network to learn fully.

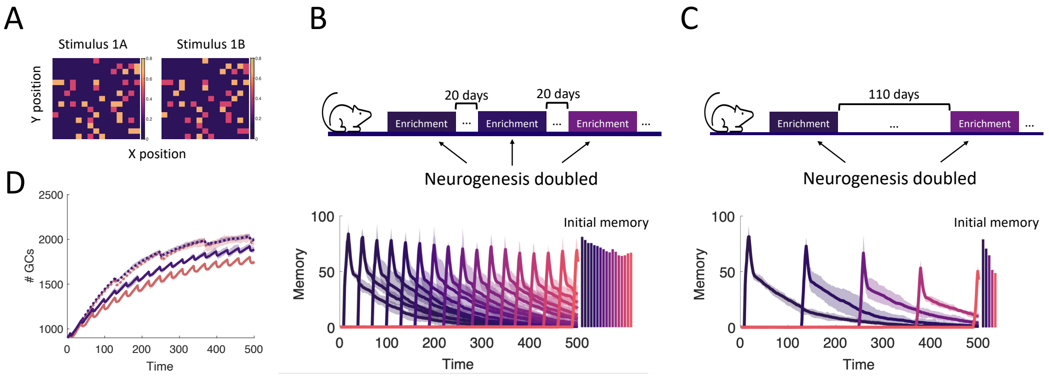


**Figure S3. (Related to Figure 4) Effects of increased abGC survival during enrichment.** (A) Example sparse, random stimuli. For each stimulus pair, 20% of MCs were randomly selected to be stimulated. Of these MCs, half were highly stimulated and half were moderately stimulated for the first stimulus in the pair. For the second stimulus, the MCs that were previously highly stimulated were moderately stimulated and those that were previously moderately stimulated became highly stimulated. This was to ensure the stimuli in the pair are difficult to discriminate. (B,C) Simulations in Figure 4D,E were repeated while doubling the number of new, fully functional abGCs on each day of enrichment to mimic the established results that olfactory enrichment increases the number of abGCs that survive until they start integrating into the network ***Rochefort et al.*** (*2002*). This functional doubling of neurogenesis slightly increases the initial memory of each enrichment (see also Figure S7D), but does not impact the prediction that more frequent enrichment improves memory. (D) The number of GCs over time for the model with a constant neurogenesis rate (orange) and with enrichment-increased neurogenesis (purple). Solid lines indicate trials with 20 day inter-enrichment intervals (orange: Figure 4D, purple: Figure S3B), dotted lines indicate trials with 110 day inter-enrichment intervals (orange: Figure 4E, purple: Figure S3C).

bioRxiv preprint doi: https://doi.org/10.1101/2024.03.03.583153; this version posted November 20, 2024. The copyright holder for this preprint (which was not certified by peer review) is the author/funder, who has granted bioRxiv a license to display the preprint in perpetuity. It is made available under aCC-BY 4.0 International license.

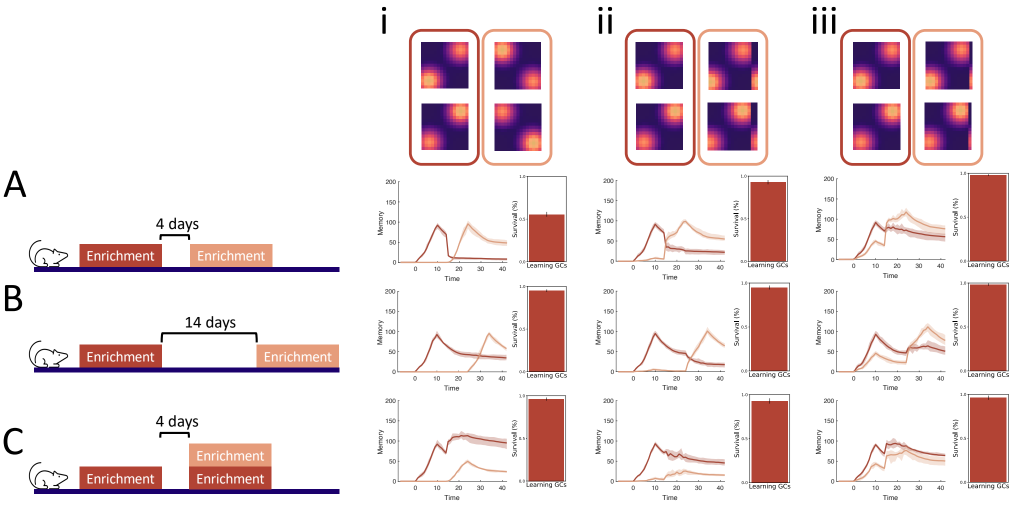


**Figure S4. (Related to Figure 4) Retrograde interference** The experiments in ***Forest et al.*** (***2019***) were simulated for different pairs of artificial stimuli. (A-C) Experimental protocols in ***Forest et al.*** (***2019***). There were two enrichment periods with two different pairs of stimuli separated by either a 4 (A,C) or 14 (B) day interval. In (C) the odors from the first enrichment were also presented during the second enrichment period in addition to the new odors. (i-iii) Enrichment stimuli. In (i) the enrichment odors were largely non-overlapping. For (ii) and (iii), moderately and highly overlapping stimuli were generated, respectively, by using for stimulus 2 correspondingly cyclically shifted versions of stimulus 1. Line plots show the memory traces resulting from the enrichment protocol marked with the corresponding color in the same row and the stimuli in the same column. Lines: mean over eight trials, shaded areas: range of values. Bar plots show the percentage of GCs that encoded the first enrichment that survived at the end of the simulation. Odor-encoding GCs were determined by clustering the connectivity of GCs at the end of the first enrichment (cf. Fig.2). Bars indicate the mean and error bars show the standard deviation. (Ai) The memory of the first enrichment was extinguished during the second enrichment and there was a significant level of apoptosis among odor-encoding GCs. (Bi) The second enrichment did not substantially affect the initial memory, and there was little apoptosis among odor-encoding GCs. (Ci) The initial memory and the GCs that encoded that memory persist through the second enrichment. (Aii) There is a significant decline in the initial memory during the second enrichment, although the odor-encoding GCs survive throughout the simulation, indicating the memory decline is a result of overwriting rather than apoptosis. (Bii) A slight memory decline occurs during the second enrichment. (Cii) The initial memory is maintained, but the network struggles to encode the second memory. (Aiii-Ciii) The second enrichment does not lead to any deficit in the initial memory, and there is no significant apoptosis.

bioRxiv preprint doi: https://doi.org/10.1101/2024.03.03.583153; this version posted November 20, 2024. The copyright holder for this preprint (which was not certified by peer review) is the author/funder, who has granted bioRxiv a license to display the preprint in perpetuity. It is made available under aCC-BY 4.0 International license.

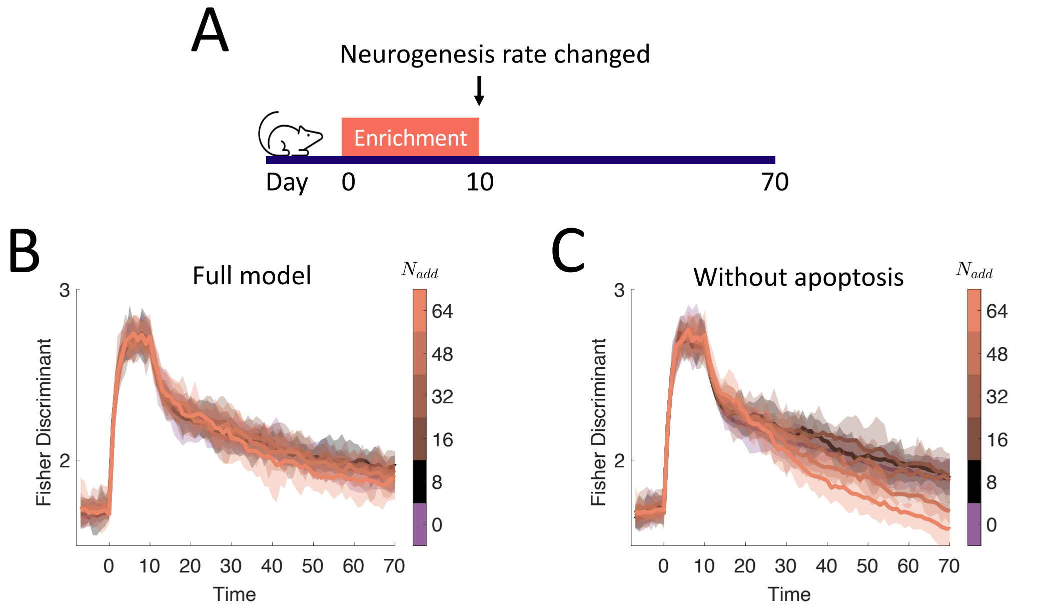


**Figure S5. Post-learning changes in neurogenesis rate.** (A) Simulation protocol. Following a 10 day enrichment (using the odors in Figure 2B), the neurogenesis rate was permanently changed. (B, C) Fisher discriminant between the two similar odors for the full model and the model without apoptosis. The Fisher discriminant was chosen in order to investigate the degree that abGCs interfere with MC activity, which represents the output of the network. The full model can tolerate the addition of vast numbers of new neurons without substantially affecting memory. Without apoptosis, the accumulation of neurons has substantial impact on memory. Lines: mean values over eight simulations. Shaded area: full range of values.

bioRxiv preprint doi: https://doi.org/10.1101/2024.03.03.583153; this version posted November 20, 2024. The copyright holder for this preprint (which was not certified by peer review) is the author/funder, who has granted bioRxiv a license to display the preprint in perpetuity. It is made available under aCC-BY 4.0 International license.

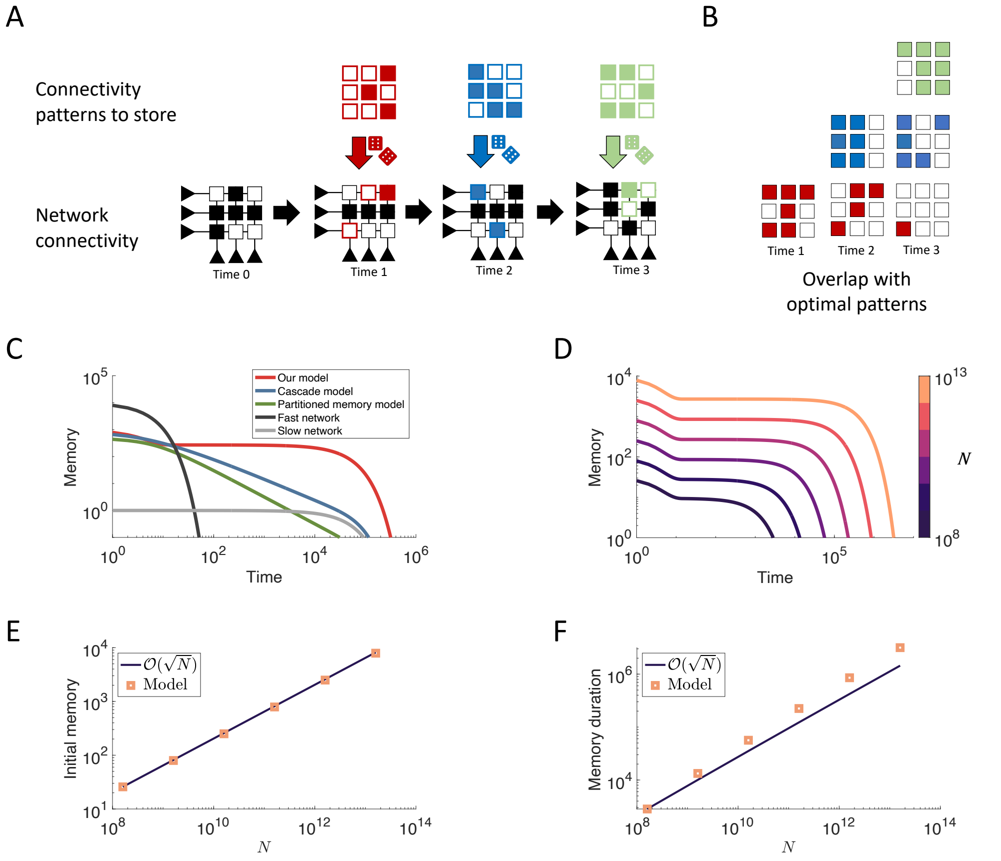


**Figure S6. Mean-field model.** (A) We assume there exists an optimal configuration that can process a given stimulus. In this framework, the network directly encodes this configuration stochastically according to the plasticity rate at each synapse, and at each time point a new stimulus is presented to the network. We track the memory of the network as the degree of overlap between the optimal network for a given stimulus and the current configuration of the network (see Supplementary Information "Comparison with other methods resolving the flexibility-stability dilemma"). Note that a lack of connection can also represent an overlap. B) Overlap between each stimulus and the current configuration of the network in (A). (C) Results of the mean-field approximation to the model described in (A) with age-dependent synaptic plasticity rates has similar initial memory and memory duration as the cascade model ***Fusi et al.*** (***2005***), and the partitioned-memory model ***Roxin and Fusi*** (***2013***). (D) Results of the age-dependent model for different values of the number of synapses $N$. (E) Initial memory as a function of $N$. (F) Memory duration as a function of $N$.

bioRxiv preprint doi: https://doi.org/10.1101/2024.03.03.583153; this version posted November 20, 2024. The copyright holder for this preprint (which was not certified by peer review) is the author/funder, who has granted bioRxiv a license to display the preprint in perpetuity. It is made available under aCC-BY 4.0 International license.

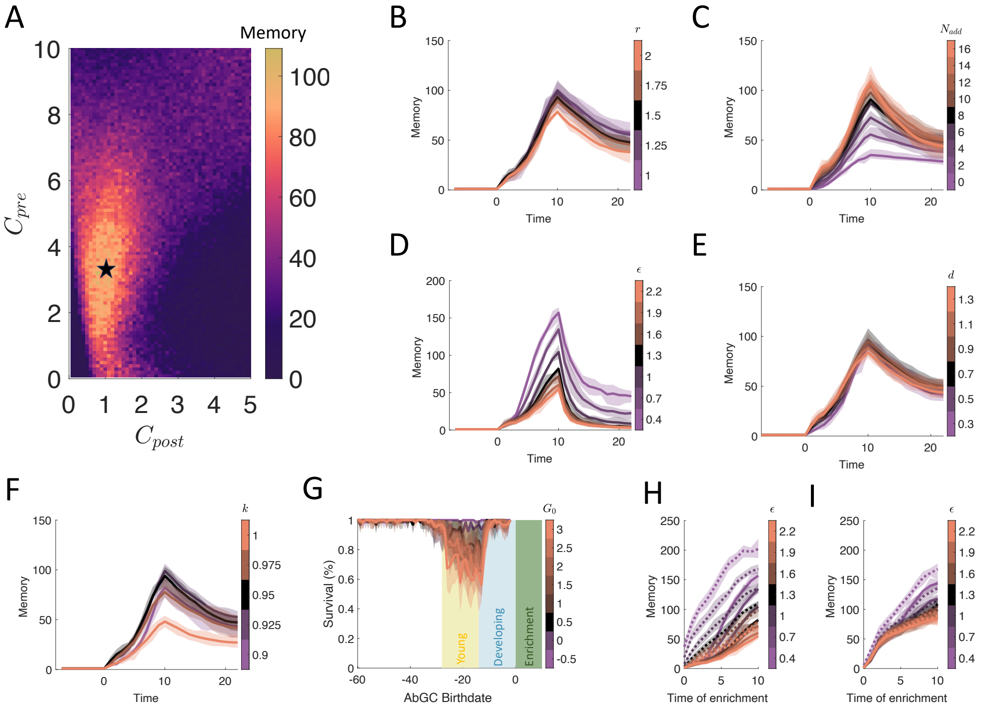


**Figure S7. Parameter sensitivity.** In all plots the values indicated in black are the parameter values used throughout this study. Lines indicate the mean and shaded areas represent the range over eight trials. (A) Final memory following the standard enrichment experiment as a function of $C_{pre}$ and $C_{post}$. (B-F) Memory trace over the course of the standard enrichment experiment for different values of $r$ (for abGCs in their critical period), $N_{add}$, $\epsilon$, $d$, and $k$, respectively. In (D), $R_0$ was increased to 1 following enrichment to illustrate the final memory value after forgetting. (G) GC survival following enrichment for different values of $G_0$ during the critical period of the abGCs (cf. Figure 4C) (H) Results of the relearning experiment (cf. Figure 3F) for different values of $\epsilon$. Solid: initial learning, dashed: re-learning. (I) As in (H) but for $N_{conn} = 60$ instead of 30.
